# The Q and A—The MIVI Q Catheters for Aspiration Thrombectomy—Initial Experience from London

**DOI:** 10.3390/jcm10245844

**Published:** 2021-12-13

**Authors:** Levansri Makalanda, Joseph Lansley, Ken Wong, Oliver Spooner, Pervinder Bhogal

**Affiliations:** 1 Department of Interventional Neuroradiology, The Royal London Hospital, Whitechapel Road, London E1 1BB, UK; joseph.lansley@nhs.net (J.L.); kenwong@nhs.net (K.W.); Paul.bhogal@nhs.net (P.B.); 2 Department of Stroke, The Royal London Hospital, Whitechapel Road, London E1 1BB, UK; o.spooner@nhs.net

**Keywords:** aspiration, thrombectomy, large-bore catheter

## Abstract

Background: Aspiration thrombectomy is a widely accepted treatment option for large vessel occlusion (LVO). The MIVI aspiration system has a novel design to maximize the lumen size. We present the results of our initial experience with this innovative aspiration thrombectomy system. Materials and Methods: Retrospectively, we reviewed our database to find all cases of LVO treated with the MIVI Q system (February 2019 and July 2020). In addition, we recorded the baseline demographics, NIHSS, ASPECT, mTICI scores, procedural time, complications, and 90 day mRS. Results: Herein, we identified 25 patients with an average age of 65.3 ± 19.3 years (range 19–89), majority of whom were female (*n* = 14, 56%). The average NIHSS was 16.9 ± 6.7 (range 6–30), and the average CT ASPECT was 7.9 ± 1.4 (range 5–10). The most common clot location was the M1 segment of the MCA (*n* = 16, 64%). Four of the patients had tandem lesions (16%). The average clot length was 21.7 ± 31 mm (range 2–130 mm). Of the 23 cases where the Q catheter reached the proximal clot face, mTICI ≥ 2b was achieved on the first pass in 11 cases (44%), and at the end of the ‘Q aspiration’ only the procedures with 16 patients achieved mTICI ≥ 2b recanalization (64%). Stent-retrievers were used in 13 cases (52%). At the end of the procedure, 24 patients (96%) achieved mTICI ≥ 2b with 18 patients (72%) achieving mTICI ≥ 2c. The average number of passes with the Q catheter, including when it was used for SOLUMBRA, was 2.1 ± 2.2 (range 1–10). The mean procedure time was 69 ± 32 mins (range 7–116 mins). No complications were associated with the MIVI Q. Conclusions: The MIVI aspiration system is a novel technology with regards to aspiration mechanical thrombectomy. The system is easy to use with early results comparable to other large-bore catheter systems. However, further studies are needed.

## 1. Introduction

The success of mechanical thrombectomy has led to a paradigm shift in the management of patients with large vessel occlusion (LVO) [1,2,3,4,5]. These trials provided level 1A evidence in support of recanalization for LVO in patients within the early time window. Subsequently, further evidence supported the same strategy for selected patients presenting up to 24 h [6,7]. The vast majority of procedures performed in these studies used stent-retrievers. However, there are a variety of different devices and techniques that can be used to recanalize occluded vessels. The principle alternative technique is direct aspiration, and this has gained increasing popularity [8]. Some authors advocate a first-line aspiration approach with bailout stent-retriever thrombectomy for failed aspiration or distal embolization with the potential promise of faster recanalization times and lower procedural costs [9]. One of the principal requirements for success with aspiration is the use of large-bore aspiration catheters. A wide variety of catheters have entered the market each with differing properties. However, they all share the same design, which resembles a long pipe. The MIVI Q aspiration catheters have a novel design with the aim of maximizing the lumen size and thereby, increasing the flow. Here, we present the results of our initial experience with this innovative aspiration thrombectomy system.

## 2. Methods

### 2.1. MIVI Aspiration System

**Figure 1:**
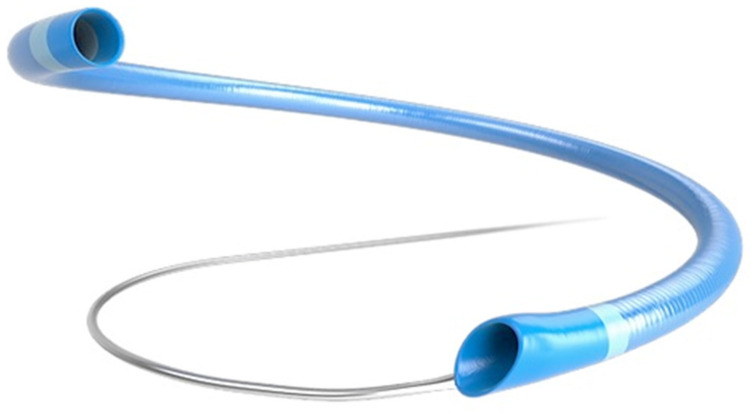
The MIVI Q system consists of a distal catheter with a flared proximal end that forms a tight seal with the selected guide catheter, and a proximal stainless steel wire replaces the proximal shaft of a traditional catheter design.

The R^4^Q aspiration catheter (MIVI Neuroscience, Inc., Eden Prairie, MN, USA) replaces the proximal catheter shaft with a long 0.018 inch stainless steel wire (Figure 1). The R^4^Q catheters all have the same outer diameter (OD) proximally (0.088 inch), with distal ODs of 0.082, 0.072, 0.055, and 0.048 inch called R^4^Q 6Fr, 5Fr, 4Fr, and 3Fr, respectively. The length of the catheters varies with the longest catheter, the Q3, which is 163 cm with a 43 cm catheter section, and the shortest, the Q6, measures 145 cm in length with a 25 cm catheter section. Similar to standard aspiration catheter designs, the R^4^Q is delivered to the clot site via a micro-wire and micro-catheter within an 0.088–0.090 inch guide catheter. Aspiration is applied directly to the guide catheter, and the total system length is variable as the R^4^Q is extended outward and retracted into the guide catheter [10].

### 2.2. Patient Selection

Retrospectively, we reviewed our maintained database to find all cases of acute ischaemic stroke (AIS) secondary to LVO with the MIVI aspiration system between February 2019 and July 2020. All of the patients underwent CT imaging of the head and CT angiography from the aortic arch to the vertex. Patients that meet the criteria for IV tPA receive thrombolytic therapy before the transfer to the angiosuite for mechanical thrombectomy, which is standard in our institution. We recorded the baseline demographic for the patients, their presenting NIHSS and ASPECT score, beginning and ending mTICI scores, time from puncture to reperfusion, and post-procedure haemorrhage. 

### 2.3. Endovascular Procedure

All of the procedures were performed via a right common femoral puncture or right radial approach using an ultrasound guidance. Through an 8Fr short sheath, an 0.088 or 0.090 inch inner diameter (ID) guide catheter was tracked into the high cervical ICA ipsilateral to the occlusion. An aspiration first policy was used in all of the cases. After angiographic confirmation of the clot location, the largest sized Q catheter thought to be able to reach the clot was selected, typically a Q5 or Q6 for M1 and ICA-T lesions and a Q3 or Q4 catheter for lesions located in the ACA or distally in M2 branches. Initially, in our experience only, the Q5 and Q6 catheters were available. However, subsequently, the Q3 and Q4 catheters also became available. Over the course of our experience, the technique used to track the Q catheter was adapted. Our initial attempts to track the Q catheters over a 0.014 inch microwire without a microcatheter or to ‘snake’ the catheter were relatively unreliable. Subsequently, a 0.014 inch microwire with a 0.027 microcatheter, was loaded into the Q catheter outside the patient and the whole system simultaneously advanced together. Whenever possible, the attempt was to reach the site of the occlusion without first crossing it with the microwire or microcatheter. After the aspiration catheter was delivered to the proximal clot face, both the microwire and microcatheter were removed, and the Q catheter advanced 2–3 mm further to fully engage the proximal clot face. After 2 min of flow arrest, the Q catheter was slowly withdrawn from the patient. If no free-flowing blood was seen during the withdrawal of the Q catheter, it was entirely removed from the guide catheter. In addition, if the clot could not be seen as trapped at the distal opening of the Q catheter, a 7Fr dilator was inserted into the proximal opening of the Q catheter. Moreover, saline was flushed and injected into the Q catheter to flush out any clot trapped within the catheter.

An additional stent-retrieval (SOLUMBRA technique) was used if there was a failure in achieving mTICI 2c or 3 or if an appropriately sized Q catheter was unavailable. The choice of stent-retriever used was based on the operator’s preference and the location of the clot. 

### 2.4. Post-Op Imaging and Care

All of the patients underwent an unenhanced CT of the head at 24 h. Haemorrhage was graded according to the Heidelberg classification [11]. All of the patients were reviewed in a clinic or via tele-clinic by a stroke neurologist to determine their mRS at 90 days.

## 3. Results

### 3.1. Baseline Demographics and Clinical Characteristics

We identified 25 patients with an average age of 65.3 ± 19.3 years (range 19–89), the majority of whom were female (*n* = 14, 56%). The majority of the patients were not eligible for IV tPA (*n* = 15, 60%). The average NIHSS was 16.9 ± 6.7 (range 6–30). The average CT ASPECT was 7.9 ± 1.4 (range 5–10). The most common clot location was the M1 segment of the MCA (*n* = 16, 64%) with five patients with clots in M2 branches (20%), two patients with ICA clots (8%), and the remaining two patients with clots in both the ACA and MCA territories (8%). Four of the patients had tandem lesions (16%). The average symptom onset time to the groin puncture time was 455 ± 410 min (range 50–1920). The average clot length was 21.7 ± 31 mm (range 2–130 mm) for the hyperdense clots seen on CT. The details are summarized in Table 1.

### 3.2. Revascularization and Endovascular Procedures

**Figure 2:**
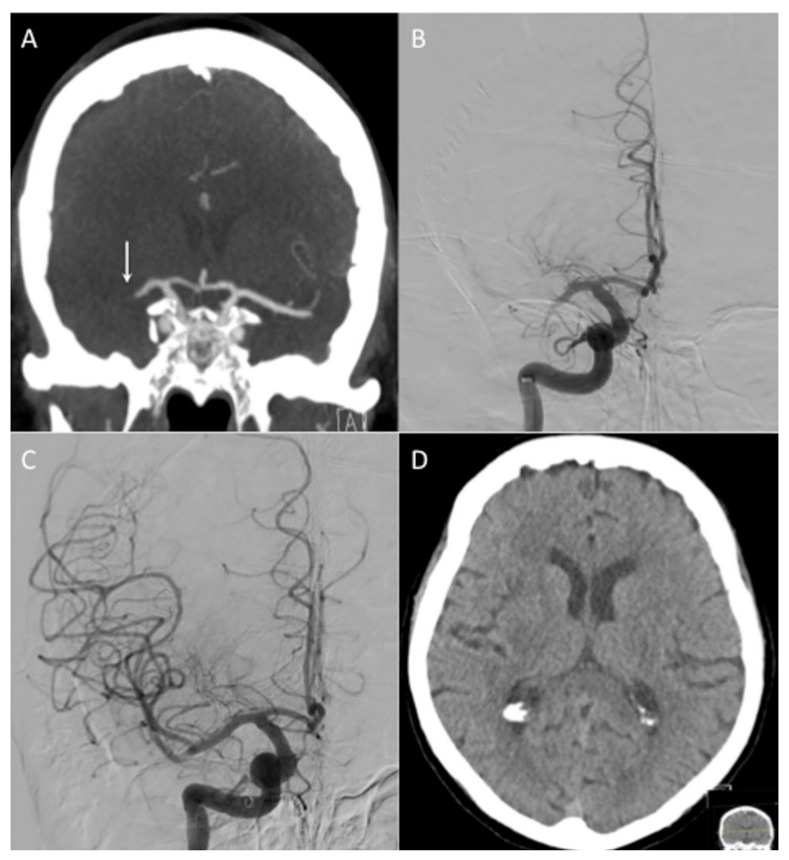
Patient 20 presented with an NIHSS of 12 and mid M1 occlusion on CT angiography (**A**, white arrow). After tracking a Ballast guide catheter (Balt Extrusion, Montmorency, France) into the high cervical ICA, angiography confirmed the clot (**B**). A Q6 catheter was ‘snaked’ to the clot face and aspiration thrombectomy was performed, resultingin TICI 3 recanalization in 1 pass (**C**). There was good preservation of grey-white matter differentiation on the 24-hCT scan with no evidence of haemorrhage (**D**). The groin puncture to recanalization time was 7 min.

The Q catheter was able to reach the clot face in 23 patients (92%). In the remaining two patients, the Q catheter was not able to reach the proximal clot due to the guide catheter instability in one case and marked vessel tortuosity in the other. Additionally, in both of these cases, alternative catheters were used. A transradial approach was used in two patients, and in the remaining 23 patients, a right femoral approach was used (Table 2). Of the 23 cases where the Q catheter reached the proximal clot face, mTICI ≥ 2b was achieved on the first pass in 11 cases (44%) and at the end of the ‘Q aspiration’, only the procedures with 16 patients achieved mTICI ≥ 2b recanalization (64%) (Figure 2). Stent-retrievers were used in 13 cases (52%). At the end of the procedure, 24 patients (96%) achieved mTICI ≥ 2b with 18 patients (72%) achieving at mTICI ≥ 2c in one affected territory, e.g., mTICI 3 in the MCA and mTICI 2c in the ACA territory are counted as mTICI 2c. The average number of passes with the Q catheter, including when it was used for SOLUMBRA, was 2.1 ± 2.2 (range 1–10). The mean procedure time, defined as puncture time to last angiographic run time, was 69 ± 32 min (range 7–116 min). Acute carotid stenting was required in three patients. Two patients were confirmed positive for SARS CorV2. There were no complications related to the procedure or Q catheters with no evidence of intra-operative vessel perforation or dissection.

On the 24 h CT head, haemorrhage was seen in four patients, but none of the patients had symptomatic intracranial haemorrhage. The 90-day mRS was available for 19 patients with eight patients recorded as mRS ≤ 2 (42%). In total, seven patients were recorded as mRS 6 (36.8%) (Table 2). 

## 4. Discussion

Aspiration thrombectomy is widely recognized as an alternative to stent-retriever mechanical thrombectomy. The proponents of aspiration highlight the relatively faster procedural times, the requirement for less equipment, and the potential reduction in cost, as well as the benefit of not requiring the crossing of the clot [9,12,13]. However, some challenges remain [14,15,16]. One of the most critical aspects of aspiration thrombectomy is the size of the aspiration catheter used, which can in turn, affect the tip force generated, and the achievable volume of aspiration. These effects will impact the ability of the aspirating catheter to ingest the clot and the success rate of aspiration. The differential effect that the catheter size has on success was demonstrated by Alawieh et al. [17]. The authors reviewed 500 stroke thrombectomies using the ‘A Direct Approach as First Pass Technique’ (ADAPT) and looked at the outcome relative to the introduction of larger bore aspiration catheters. They showed that the likelihood of successful recanalization with an aspiration only was significantly higher when the ACE 064 and ACE 068 catheters (Penumbra) were used relative to the 5MAX (Penumbra) (80.5% and 84.9% vs. 61.1%, *p* < 0.05). However, there was no significance between the ACE 060 and 5MAX catheters. The use of the ACE 068 and 064 catheters was associated with a significantly lower likelihood of requiring a stent-retriever as bailout (27.8% and 29.3% vs. 41.8%, *p* < 0.05), as well as shorter procedure times compared with the 5MAX (*p* < 0.05) catheter. There was no significant difference in the intraprocedural complication rate or the rates of PH-2 haemorrhage across the different devices. However, the use of the ACE 068 catheter was an independent predictor of functional outcome (mRS ≤ 2) at 90 days (OR = 1.6, *p* < 0.05). The authors noted that ‘ingestion’ of the clot en-bloc was more likely to be achieved when the larger bore aspiration catheters were used. In addition, this had the advantage of potentially limiting the likelihood of clot fragmentation, which could occur if the clot is simply ‘engaged’ with the distal catheter tip and removed slowly through the guide catheter. The additional advantage of the ingestion is that the catheter remains in situ should a bailout stent-retriever thrombectomy be required. 

Several large-bore catheters are available on the market. A recent publication by Almallouhi et al. [18] reported on the results of the AXS Vecta (Stryker Neurovascular, Fremont, CA, USA), which was the first 0.071-inch catheter approved for mechanical thrombectomy. In total, 10 patients underwent mechanical thrombectomy using an ADAPT first-line approach. The AXS Vecta was delivered to the site of the occlusion over a 0.016 inch microwire and microcatheter. Of note, six of the clots were located in M2 branches with three M1 occlusions and a single tandem M1 lesion. In total, nine cases resulted in TICI ≥ 2b reperfusion between 1 and 3 attempts. In the only case that resulted in TICI 2a reperfusion, the authors reported an underlying atherosclerotic plaque. Moreover, despite the efforts with both aspirations and stent-retrievers, a successful reperfusion was not achieved. Satti et al. [19] published the results of their multicenter study which looks at the 0.071 and 0.074 Vecta AXS catheters. Across two sites and 26 patients (18 treated with 0.071 Vecta and eight treated with 0.074 Vecta), direct aspiration was used as a first-line technique in all of the cases and was successful in 72% of the cases. In the remaining 28% of cases, the use of a stent-retriever to deliver the aspiration catheter or for rescue thrombectomy (direct aspiration unsuccessful or to target residual small, distal emboli) was required. Similar promising results from other large-bore aspiration catheters have also been recently reported [20,21].

Torabi et al. [22] reported their initial experience using the R^4^Q in 32 consecutive patients with LVOs in both the anterior and posterior circulation. The MIVI catheter could be successfully delivered to the site of the occlusion in 87% of patients (*n* = 28). Successful recanalization (mTICI ≥ 2b) on the first pass was achieved in 65% of patients (21/32) and 62% of patients (16/26), where the R^4^Q was successfully delivered without a stent-retriever. A final mTICI ≥ 2b was achieved in 29/32 (91%) patients and 26/28 (93%) patients, where the R^4^Q was successfully delivered. Interestingly, the authors compared the first pass results with the ASTER and COMPASS trial results. The first pass of mTICI ≥ 2b with MIVI was more frequent than in ASTER (62% vs. 26%, *p* = 0.0009) and equivalent to COMPASS (62% vs. 57%, *p* = 0.83). In addition, it was more frequent in comparison to the combined ASTER and COMPASS cohort (62% vs. 40%, *p* = 0.04). In our series, mTICI ≥ 2b was achieved in 45% of patients with bailout techniques needed in 54% of cases. We believe that these results may at least, in part, be due to the fact that we did not have a full complement of the Q catheters at the start of the study. Therefore, we were not able to attempt aspiration with the Q catheters for distal clots. 

Early studies that sought to determine the tip force generated by different catheters demonstrated that the force was proportional to the surface area of the catheter tip [23,24]. This principle is governed by the Hagen–Poiseuille Law. This essentially states that the flow and pressure are related to several factors, including the viscosity of the fluid, as well as the length and diameter of the tube. In this case, the diameter has the greatest effect since it alters to the power 4, whereas the others alter the flow rate linearly. Therefore, the design of standard catheters is limited by the internal diameter of the catheter, as well as the length. The design of the R4Q aspiration catheter (MIVI Neuroscience, Inc., Eden Prairie, MN, USA) overcomes these limitations by replacing the proximal three-quarters of the catheter shaft with a 117 cm, 0.018 inch stainless steel control wire and enabling the utilization of the internal area of the guide catheter for the majority of its length. As a result, the aspiration is partly delivered via the guide catheter, which can be up to 0.090 inches ID, and via the R4Q catheters with suction applied directly to the guide catheter. The total system length is variable as the R4Q is extended outward and retracted into the guide catheter. In addition, this will alter the tip force with greater suction, which is applied to the tip as the catheter is withdrawn into the guide catheter and the system is ‘shortened’. Long et al. [10] sought to quantify the different tip forces and aspiration capabilities of the R4Q system in comparison to other commonly used aspiration catheters. The authors showed that for every size of an aspiration catheter tested, from 3–6Fr, there was a significant increase in flow rate compared with other similarly sized catheters with increases of 21.9%, 24.7%, 61.9%, and 244.7% for the R4Q 6Fr, 5Fr, 4Fr, and 3Fr systems, respectively. Moreover, this flow rate increases on retraction of the catheter into the guide catheter. Similarly, the 6Fr R4Q catheter produced a tip force of 140.2% higher than other catheters of similar size. Similarly, the use of proximal occlusion may reduce the pressure gradient across the clots and improve the results from aspiration [25]. Another potential advantage of the design of the Q catheters is their gradually increasing proximal diameter, which is unlike the design of standard aspiration catheters that are essentially of uniform diameter along their entire length. This increasing diameter will allow a gradual increase in the aspiration force once the clot has passed the distal end of the catheter, which is the narrowest part of the catheter. As a result, the force of aspiration which is applied to the clot within the catheter will also be maintained and actually increase with the decrease of friction upon the clot within the larger diameter parts of the catheter. These factors may improve the complete ingestion of certain clot types, especially if they are deformable, and improve recanalization.

Recently, a study by Kang et al. [26] analyzed data from a multicenter registry in Korea to compare the outcome of aspiration thrombectomy performed using a balloon guide catheter (BGC) or a conventional guide catheter. A total of 429 patients were enrolled, with a BGC used in 45.2% of patients. The use of a BGC significantly reduced the number of aspiration passes (mean, 2.6 vs. 3.4; *p* < 0.001), puncture-to-recanalization time (mean, 56 vs. 64 min; *p* = 0.018), and embolization to distal or different site (0.5% vs. 3.4%, *p* = 0.045). The BGC group showed significantly higher final (89.2% vs. 72.8%, *p* < 0.001) and first-pass recanalization success rates (24.2% vs. 8.1%, *p* < 0.001). In our series, the Q catheters were not used in conjunction with a BGC due to the restraints on ID size. The advent of large ID BGCs, such as the Walrus (Q-Apel medical, Fremont, CA, USA) will enable the Q catheters to be used with a BGC. We believe this will improve both the first pass success rate and the overall success rate as suggested by other studies combining aspiration with BGC [27].

There is still an ongoing debate as to which method, aspiration or stent-retriever, offers the optimal strategy for mechanical thrombectomy. Proponents of the aspiration technique point to its faster recanalization times and cost effectiveness, whereas detractors point out that trials using aspiration have shown good recanalization rates. However, these do not translate into improved mRS scores at 90 days. The reason for this is thought to be due to the greater number of distal emboli when aspiration is used alone [14,28]. Chueh et al. [28] assessed the risk of distal embolization with SR and aspiration using the Solitaire (Medtronic), 5MAX (Penumbra) with and without the additional use of balloon guide catheters. They showed that with soft elastic clots the use of a BGC significantly reduced the risk of distal emboli compared with the use of a standard guide catheter. They also showed that ADAPT resulted in the most emboli overall for both soft elastic clots and hard clots with the vast majority of these clots as very small (<50 μm). These very small emboli could result in a large vascular territory which is affected, and many micro-infarcts that result in a poorer outcome. This problem is likely compounded by the fact that most of the BGCs currently cannot accommodate the larger bore aspiration catheters, and thus the anterograde flow arrest cannot be achieved. Even when a combined technique is used, it should be borne in mind that distal fragments can be dislodged when the SR is unsheathed if there is no proximal flow control. For this reason, it may be prudent to temporarily inflate the BGC and/or track the aspiration catheter to the proximal clot face with the aspiration on at the time of SR deployment. This has recently been shown to minimize the number of distal emboli in an in vitro model [29]. Similarly, it is important that aspiration catheters are positioned correctly as the inappropriate positioning of the aspiration catheter proximal to the clot actually results in arterial collapse [30]. Theoretically, a larger bore catheter may help with some of these potential issues, especially if compatible with the newer larger bore BGCs. However, this is yet to be determined in prospective studies. The majority of clots in our study were hyperdense and these clots are thought to be erythrocyte rich rather than fibrin rich, resulting in softer, more elastic clots rather than firmer more challenges clots to remove. The optimization of techniques based on imaging correlates of underlying clot architecture is something that is actively pursued alongside newer devices designed to analyze the clot architecture and composition in real time. 

Our study is limited by its small size and retrospective nature, as well as lack of core lab evaluation. In the early phase of our experience with the Q catheters, the smaller Q catheters (Q3 and Q4) were not available. Therefore, in the case of distal clot migration, a stent-retriever was required. 

## 5. Conclusions

The MIVI Q catheters are a novel technology with regards to aspiration mechanical thrombectomy. They are easy to use with results comparable with other large-bore catheter systems. Further studies are required, including the use of the Q catheters with large-bore BGCs.

## Figures and Tables

**Table 1:** Baseline demographics, clinical, and imaging data.

	Demographic			Pre-Op							Onset to Puncture (min)
	Gender	Age	TPA	Baseline mRS	NIHSS	CT ASPECTS	Clot Location on CTA	Hyperdense Clot	Clot Length	Tandem Lesion (Y/N)	
1	F	70	N	3	11	8	M1 (stenosis)	N	NA	N	395
2	F	74	Y	1	19	8	M1	Y	13	N	300
3	F	67	N	0	13	8	M1	Y	12	Y	165
4	M	19	Y	0	18	6	Petrous ICA	Y	130	N	525
5	F	89	N	1	22	10	M1	Y	8	N	1920
6	M	71	Y	0	20	10	M1	Y	20	Y	168
7	F	84	Y	3	30	8	M2 and A3	Y	2	N	165
8	F	89	N	1	18	9	M2	Y	2	N	465
9	F	46	N	3	19	9	M2 and A2	Y	14	Y	330
10	M	70	N	3	23	8	M1	N	NA	N	150
11	F	26	N	0	7	8	M2	Y	6	N	296
12	F	58	N	0	24	5	M1	Y	10	N	217
13	F	43	Y	0	22	8	M1	N	NA	N	420
14	F	84	N	0	8	8	M2	N	NA	N	825
15	M	77	N	0	24	6	ICA T	Y	30	N	570
16	M	60	N	0	27	5	M1	Y	70	Y	390
17	M	74	N	0	6	8	M2	Y	9	N	453
18	M	60	Y	0	24	8	M1	Y	14	N	186
19	M	44	Y	0	17	9	M2	N	NA	N	160
20	F	88	N	1	12	7	M1	Y	10	N	535
21	M	50	Y	0	12	8	M1	Y	20	N	180
22	F	88	Y	0	16	9	M1	N	NA	N	365
23	M	56	N	0	14	6	M1	Y	13	N	900
24	F	83	N	0	9	9	M1	N	NA	N	1260
25	M	62	Y	0	9	9	M1	Y	8	N	50

**Table 2:** Procedural data, recanalization results, complications, and follow-up data.

	Equipment/Procedure										
	Guide Catheter	MIVI Catheter Used	1st Pass mTICI	mTICI at End of MIVI Procedure	Number of MIVI Passes	Bailout Stent-Retriever	Final TICI	Treatment Time (Puncture to Final Angio/Min)	ICH	90 Day mRS	Comments
1	Neuron Max	Q6	1	1	1	Solitaire implanted for ICAD	2b	90	0	5	Stenosis in M1
2	Neuron Max	Q6	2a	2a	1	Y	2b	111	3a & 3c	6	
3	Neuron Max	Q6	3	3	1	N	3	57	0	1	Acute carotid stenting
4	Neuron Max	Q6	0	2b	10	Y (A3)	2c	95	0	6	SR for ACA clot
5	Neuron Max	Q5	1	1	1	Y	3	68	0	6	
6	Neuron Max	Q6	3	3	1	N	3	28	0	1	Acute carotid stenting
7	Neuron Max	Q3	3 (M2)	3	1	Y (A3)	3	40	0	3	SR for ACA clot
8	AXS Infinity	Q5	1	1	1	Y	3	37	0	6	
9	AXS Infinity	Q3	0	0	1	Y	3 (MCA), 2c (ACA)	116	0	3	Acute carotid stenting
10	Neuron Max	Q6	3	3	1	N	3	26	0	3	
11	Neuron Max	Q6	2c	2c	1	N	2c	29	0	2	
12	Neuron Max	Q6/Q4	2c (M2)	2c	2	Y (A2)	2c	62	0	4	SR for ACA clot
13	Neuron Max	Q6	1	2b	4	N	2b	71	3c	1	
14	AXS Infinity	Q3	2b	2b	1	N	2b	26	0	1	Transradial approach
15	Fubuki	Q6/Q4	1	1	2	Y	2c	111	1a	6	Transradial approach
16	MIVI S90	Q6/Q4	0	2b	6	N	2c	75	0	4	
17	MIVI S90	Q6	Q did not reach clot	NA	0	N	2c	55	0	2	Unable to reach clot due to instability of S90
18	MIVI S90	Q6	1	2a	2	Y	2c	37	0	0	COVID +VE
19	Fubuki	Q5	2a	2b	2	N	2b	33	0	0	COVID +VE
20	Ballast	Q6	3	3	1	N	3	7	0	NA	
21	Ballast	Q6	2c	2c	1	N	2c	35	0	NA	
22	Ballast	Q6	Q did not reach clot	NA	0	Y	2c	78	0	NA	Unable to reach clot due to vessel ectasia
23	Ballast	Q6/Q4	2a	2b	4	Y	2b	109	2 & 3b/c	NA	
24	Ballast	Q5	3 (M1), 1 (M2)	3 (M1), 1 (M2)	2	Y	2a	67	3c	NA	ICAD in M2 branch
25	Ballast	Q6	3	3	1	N	3	14	0	6	Patient had underlying undiagnosed malignancy
